# Role of Mindin in Diabetic Nephropathy

**DOI:** 10.1155/2011/486305

**Published:** 2011-12-26

**Authors:** Maki Murakoshi, Tomohito Gohda, Mitsuo Tanimoto, Kazuhiko Funabiki, Satoshi Horikoshi, Yasuhiko Tomino

**Affiliations:** Division of Nephrology, Department of Internal Medicine, Juntendo University Faculty of Medicine, Tokyo 113-8421, Japan

## Abstract

A number of studies have shown that proinflammatory cytokines have important roles in determining the development of microvascular diabetic complications, including nephropathy. Inflammatory biomarkers should be useful for diagnosis or monitoring of diabetic nephropathy. Mindin (spondin 2) is a member of the mindin-/F-spondin family of secreted extracellular matrix (ECM) proteins. Recent studies showed that mindin is essential for initiation of innate immune response and represents a unique pattern-recognition molecule in the ECM. Previously, we demonstrated that the levels of urinary mindin in patients with type 2 diabetes were higher than those in healthy individuals. We propose that urinary mindin is a potent biomarker for the development of diabetic nephropathy.

## 1. Introduction

Diabetic nephropathy is a major cause of end-stage kidney disease (ESKD) in the United States, Japan, and most of Europe [1]. Although the etiology of this insidious disorder is not well understood, hyperglycemia and hypertension may play pivotal roles in the pathogenesis of diabetic nephropathy. Actually, almost 30% of diabetic patients develop diabetic nephropathy despite strict blood glucose and/or blood pressure control [2]. Chronic low-grade inflammation (so-called microinflammation) has been found to play roles in the pathogenesis of diabetes [3, 4] and has been identified as a risk factor for the development of diabetes [5, 6]. Moreover, diabetes has been proposed as a disease of the innate immune system [7]. In addition, the studies in recent years have shown that inflammation and inflammatory cytokines are determinants in the development of microvascular diabetic complications such as neuropathy, retinopathy, and nephropathy [8–11]. In 1991, Hasegawa et al. reported that glomerular basement membranes from diabetic rats induced significantly greater amounts of tumor necrosis factor-*α* (TNF-*α*) and interleukin-1 (IL-1) in cultured peritoneal macrophages than when these cells were incubated with basement membranes from nondiabetic rats [12]. Based on these findings, the authors suggested for the first time that inflammatory cytokines may participate in the pathogenesis of diabetic nephropathy [12]. At present, a number of clinical studies have suggested relationships between inflammatory cytokines and diabetic nephropathy [13, 14]. Inflammatory cytokines, that is, IL-1, interleukin-6 (IL-6), and interleukin-18 (IL-18) [15, 16], vascular endothelial growth factor (VEGF) [17, 18], monocyte chemoattractant protein-1 (MCP-1) [19, 20], and transforming growth factor-*β* (TGF-*β*) [21], as well as TNF-*α* [22–24], are involved in the development and progression of diabetic nephropathy. It has been demonstrated that endothelial, mesangial, glomerular, and tubular epithelial cells can synthesize inflammatory cytokines, which have significant renal effects. 

To survey the glomerular gene expression profile in type 2 diabetes, we performed a microarray analysis using isolated glomeruli from diabetic KK/Ta mice [25]. We observed significant differences in the expression of immune response and inflammatory-related genes. Among them, mindin (also called spondin 2, SPON2) mRNA was significantly upregulated. The present paper focuses on mindin as a urinary marker in diabetic nephropathy.

## 2. Molecular Structure of Mindin

Mindin is a highly conserved extracellular matrix (ECM) protein that is abundantly expressed in the spleen and lymph nodes [26]. It is a member of the mindin-F-spondin (FS) family of secreted ECM proteins that also includes mammalian FS, zebrafish mindin 1 and mindin 2, and *Drosophila* M-spondin [32–37]. Mindin was initially identified in zebrafish and was observed to accumulate selectively in the basal lamina [33]. Subsequently, the genes encoding rat and human mindin were cloned [35, 36]. Mouse Spon2 cDNA encodes an open reading frame of 330 amino acids with a calculated molecular mass of 36 kDa. Rat mindin is a secreted protein that promotes adhesion and outgrowth of hippocampal embryonic neurons *in vitro* [35]. All members of the mindin-FS family share three structural domains. Two domains, FS1 and FS2 (for F-spondin), are unique to this family. A third domain, called thrombospondin-type 1 repeats (TSRs), is found in a large group of proteins including thrombospondins, the semaphoring 5 family, and the ADAM (disintegrin and metalloproteinase) protein family [38]. The crystal structure of the FS domain of human mindin was demonstrated to be the domain that mediates integrin binding [39]. Remarkably, mindin also functions as a pattern recognition molecule for microbial pathogens [26] and as an integrin ligand for inflammatory cell recruitment and T-cell priming [27, 28, 30].

## 3. Role of Mindin in Inflammatory Disease

As shown in Table 1, mindin has been studied as essential factor in immune response. He et al. measured serum concentrations of TNF and IL-6 to assay cytokine concentrations in mindin-deficient mice treated with lipopolysaccharide (LPS) [26]. The serum concentrations of these cytokines in control mice treated with LPS were substantially increased. In contrast, the serum concentrations of these cytokines in the mindin-deficient mice were only slightly increased after LPS challenge, indicating that mindin is essential for the *in vivo* production of inflammatory cytokines during endotoxin-induced septic shock. Macrophages and mast cells from mindin-deficient mice show defective responses to a broad spectrum of microbial stimuli. Mindin recognizes LPS through its TSR domain [39]. Mindin also functions as an opsonin for macrophage phagocytosis of bacteria [26]. Mice lacking mindin exhibit defective clearance of influenza virus, whereas mindin-deficient macrophages show impaired activation following influenza infection [29]. Thus, mindin is a pattern recognition molecule that is critical for initiating innate immune responses such as Toll-like receptor 4 (TLR4) [29, 40] (Figure 2). Guleng et al. demonstrated that mRNA expression of mindin is upregulated during dextran sulfate sodium-induced acute intestinal inflammation and is also upregulated by CpG-ODN (a known TLR-9 ligand) stimulation *in vitro.* Moreover, mindin induces nuclear-factor- (NF-) *κ*B promoter activation in a TLR-9 mediated manner [31].

As mentioned above, our microarray data suggested a relationship between mindin and diabetic nephropathy. We focused on mindin expression in the glomeruli and attempted to determine whether an increase in urinary mindin was associated with the development of diabetic nephropathy.

## 4. Mindin and Diabetic Nephropathy

**Figure 1:**
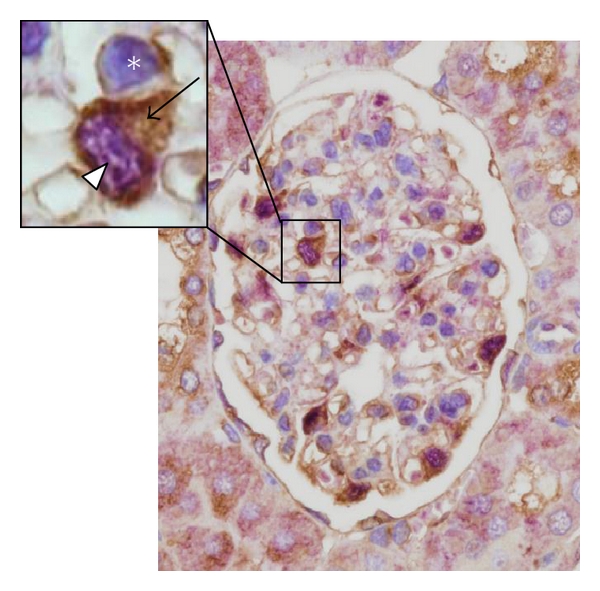
Mindin protein expression in glomeruli of 20-week-old KK/Ta mice. Double-immunohistochemical staining for mindin (brown; arrow) and WT1 (purple; arrowhead, podocyte nucleus marker) in kidney sections from mice with HD at 20 weeks of age. Counterstaining with hematoxylin (blue; asterisk) was used to visualize nuclei.

**Figure 2:**
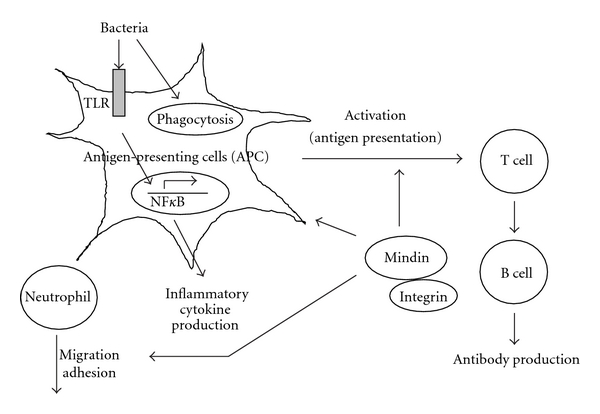
Mindin as an essential factor in immune response. Mindin has an important role in both innate and adaptive immunity.

**Figure 3:**
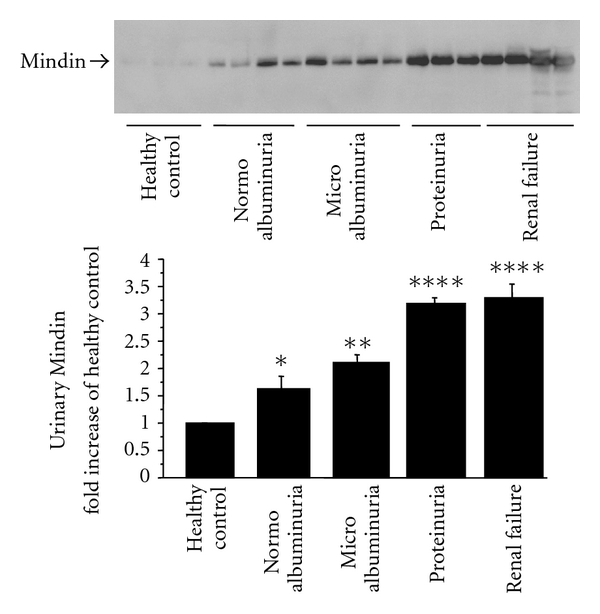
Urinary mindin secretion in patients with diabetic nephropathy by western blot analysis. Healthy control (*n* = 3), Normoalbuminuria (*n* = 4): <30 mg albumin/g creatinine, Microalbuminuria (*n* = 4): 30–300 mg albumin/g creatinine, Proteinuria (*n* = 3): >300 mg albumin/g creatinine, Renal failure (*n* = 4); ****P < 0.0001 versus healthy control; **P < 0.01 versus healthy control; *P < 0.05 versus healthy control.

In contrast to the immune reaction in response to acute infection or inflammation, the immune processes in chronic diseases such as diabetic nephropathy can be smoldering processes that are difficult to detect. Development of a diagnostic test, more convenient and reliable than those currently used, would therefore be highly desirable. Urine is easily accessible, and therefore, as a diagnostic medium allows for noninvasive detection of biomarkers. With 70% of urinary proteins originating from the kidney and urinary tract, and 30% being filtered by the kidney, urinary biomarkers are likely to be linked to renal dysfunction or systemic changes [41]. Urinary albumin, the current gold standard, is known that significant structural changes have already appeared even at the stage of microalbuminuria in type 2 diabetes patients. Thus, it is necessary to develop a more sensitive measurement for detecting the early stage of renal injury in patients with diabetic nephropathy. Inflammatory markers should be useful biomarkers for diagnosis or monitoring of diabetic complications, particularly kidney disease. However, the sensitivity of these markers compared with albumin requires further investigation. We dissected isolated glomeruli from three 20-week-old KK/Ta mice fed a high-calorie diet (HC) or a standard diet (SD). The inbred mouse strain KK/Ta, established as a diabetic strain in Japan, spontaneously exhibits characteristics of type 2 diabetes associated with fasting hyperglycemia, glucose intolerance, hyperinsulinemia, mild obesity, dyslipidemia, and albuminuria. Renal lesions in KK/Ta mice closely resemble those in the early stage of human diabetic nephropathy. The albumin/creatinine ratio (ACR) in male KK/Ta mice at 16 weeks of age is 150–200 mg/gCr. KK/Ta mice glomeruli show diffuse hyperplasia in the glomerular mesangial areas with mild mesangial cell proliferation [42]. Immunohistological studies show an intense, specific fluorescence for albumin and *γ*-globulin along the glomerular capillary walls. Therefore, KK/Ta mice are considered to provide a suitable model for type 2 diabetes and the early stage of diabetic nephropathy in humans. Mindin expression was examined using real-time PCR, western blot analysis, and immunohistochemical staining of the glomeruli (Figure 1), as well as cultured podocytes and urine samples of both mice and humans. The mindin protein expression levels in mice were localized in the podocytes, and their levels in the glomeruli were increased in the HC group compared with the SD group [25]. We also examined podocyte cultures incubated in high glucose (HG). Mindin was detected in podocyte culture supernatants and its expression levels under HG stimulation were significantly higher than those under normal glucose stimulation. Therefore, we examined the urinary secretion of mindin. Urinary mindin expression levels in the HC group were already significantly higher than those in the SD group at 16 weeks of age. The urinary mindin level seems to be increased earlier compared with the ACR. Furthermore, mindin was also detectable in human urine. Urinary mindin expression in patients with type 2 diabetes increased compared with that in healthy individuals, reflecting the stage of diabetic nephropathy (Figure 3).

**Figure 4:**
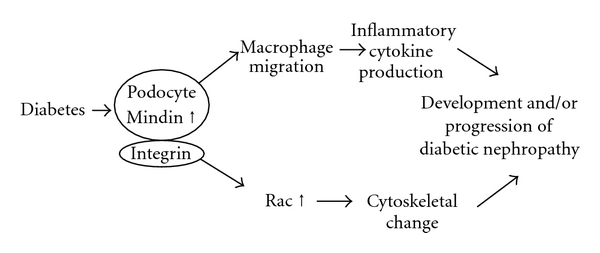
The hypothesis concerning the role of mindin in diabetic nephropathy. Mindin might be related to podocyte injury in diabetic nephropathy and regulate not only inflammation but also cytoskeletal change via integrin.

Podocytes cover the outer aspect of the glomerular basement membrane (GBM) and form the final barrier to protein loss [43]. The podocyte foot process is fixed to the GBM via *α*_3_ *β*_1_ integrin and *α*/*β* dystroglycans, and *α*_3_ *β*_1_ integrin is the major integrin expressed by podocytes [44–46]. Jia et al. reported that mindin serves as an integrin ligand [27]. We also found that *β*_1_ integrin protein expression increased in the cultured podocytes stimulated under HG conditions (data not shown). Moreover, mindin was reported as a key regulator of Rho GTPase expression. Signaling through integrins activates Rho GTPases [28]. Cytoskeletal changes of podocytes regulated by the Rho family are critically involved in the pathogenesis of glomerular diseases. Mindin should be produced by damaged podocytes under high glucose conditions and serve as a biomarker of the progression of diabetic nephropathy (Figure 4).

## 5. Conclusion

Increasing evidence indicates that inflammatory and immune response mechanisms may contribute significantly to the development and progression of diabetic nephropathy. Recent studies showed that mindin is essential for the initiation of immune response and represents a unique pattern-recognition molecule in the ECM. We focused on the role of mindin and examined the urinary secretion of mindin in mice and humans. Mindin could be an early biomarker of the progression of diabetic nephropathy.

## Figures and Tables

**Table 1:** Roles of mindin in immune response and inflammation.

Authors	Year	Role of mindin	Reference
He et al.	2004	Pattern recognition molecule for microbial pathogens	[26]
Jia et al.	2005	Integrin ligand for inflammatory cell recruitment	[27]
Li et al.	2006	Regulating Rho GTPase expression in DCs and DC priming of T lymphocytes	[28]
Jia et al.	2008	Immune-enhancing agent in influenza infection	[29]
Li et al.	2009	Trafficking of normal eosinophils into the airspace and the pathogenesis of allergic airway disease	[30]
Guleng et al.	2010	Activation of NF-kappaB in a TLR-9 mediated manner during colitis	[31]
